# DNA integrity in diagnosis of premalignant lesions

**DOI:** 10.4317/medoral.24287

**Published:** 2020-12-19

**Authors:** Noha Adel Azab, Fat'heya M. Zahran, Ayman Abdel Wahab Amin, Normeen Hany Rady

**Affiliations:** 1 Oral Medicine and Periodontology Department, Faculty of Dentistry, Cairo University, Egypt; 2 National Cancer Institute, Cairo University, Egypt; 3 Clinical and Chemical Pathology Department, Faculty of Medicine, Cairo University, Egypt

## Abstract

**Background:**

Carcinogenesis is a dynamic process which traditional biopsying can not keep up with. Saliva as fluid in the vicinity of the tumor can offer better insights to this process. This study aimed to identify the accuracy of salivary DNA integrity index in differentiating between oral premalignant lesions and oral cancer.

**Material and Methods:**

This phase II diagnostic test accuracy study included 93 patients divided into three groups: 30 oral cancer patients, 33 patients with oral premalignant lesions divided into 21 oral lichen planus patients and 12 patients with leukoplakia and 30 normal individuals who acted as controls. Oral rinse was collected from all participants and they all underwent conventional visual and tactile examination, and patients with oral lesions had the diagnosis confirmed by histopathological examination of tissue biopsy. DNA integrity index was determined as the ratio between ALU247 and ALU115 measured by qPCR.

**Results:**

There was no statistically significant difference regarding ALU115, ALU247 and DNA integrity index between the three study groups. The index was significantly higher in the oral cancer group than the oral lichen planus patients, while no significant difference was found between the oral cancer and the leukoplakia cases. The DNA integrity index sensitivity, specificity, positive and negative predictive values were 73%, 45%, 55% and 65% respectively.

**Conclusions:**

Salivary DNA integrity index showed poor diagnostic abilities in differentiating between the oral cancer and premalignant lesions.

** Key words:**DNA integrity index, oral lichen planus, leukoplakia, saliva, cell free DNA, oral cancer.

## Introduction

Oral cancer is ranked as one of the top 10 most common cancers in the world. According to the GLOBOCAN 2018, when oropharynx is added as a second site, oropharyngeal cancer represents 2.5% of all cancers, and is responsible for 228,389 deaths reported in 2018 (1). Survival rates for oral squamous cell carcinoma are highly stage dependent (2). Approximately 70% of all new cases are diagnosed at a late stage, which greatly affects their prognosis, underscoring the importance of early detection and prevention (3). Up to 70% of cancers are preceded by oral premalignant or potentially malignant disorders (OPMDs) (2). Dysplastic lesions can potentially progress into cancer within two to five years or even much later (4).

In 2017, the American Dental Association stated that the main gold standard for screening and detection of oral cancer precursor lesions remains the clinical visual examination while, microscopic evaluation of a representative sample is the gold standard for diagnosis of OPMDs (3). Although there are many adjunctive non-invasive tests that are available to the clinician, none have reported higher sensitivity or specificity than analysis of tissue biopsy. There are still no dynamic clinical tests that can distinguish between progressive and non-progressive OPMD (5).

Cancer biomarkers have been identified in both blood and saliva. As tumor markers and metabolites are released into the vicinity of the tumor, saliva seems to be an ideal medium for markers of oral cancer, particularly as it is accessible and non-invasive (6).

Cell-free DNA (cfDNA) is physiologically seen in humans. It can be nuclear or mitochondrial in origin and is found in almost all extracellular substances such as blood, saliva, urine, milk, lymph, bile, spinal fluid and amniotic fluid (7).

Tumerogenesis includes multiple somatic alterations, thus, multiple samples are required for adequate tumor characterization, which is difficult to achieve with the current diagnostic standard- the tissue biopsy. cfDNA can be an alternative solution for tumor characterization especially when a tumor is inaccessible or the patient is too frail to operate. Tumor cfDNA has shown more than 80% concordance with tumor tissue suggesting that in the future it can provide a more comprehensive tumor profile than the regular biopsy (8).

Apoptosis is the main source of cfDNA in healthy individuals. It produces uniform small fragments of neatly digested DNA. Necrosis - a process enhanced by malignancy- produces incompletely digested DNA fragments that are mostly long. The varying lengths of DNA fragments are used to calculate DNA integrity index which is the ratio between long and short strands. (9)

The short interspersed elements amplicons ALU115 and ALU247 are the most commonly employed form of DNA integrity assessment and have high species specificity. ALU115 primer binding sites are within the ALU247 and can amplify both longer and shorter strands. So, ALU247 is an indicator of necrotic cell death, while ALU115 is an indicator of overall cell death (10).

This study aimed at identifying the diagnostic accuracy of salivary DNA integrity index and its ability to differentiate between oral premalignant lesions and oral cancer.

## Material and Methods

This is a prospective phase II diagnostic test accuracy study. Its protocol is registered on clinicaltrials.gov under identifier NCT03682562 (https://clinicaltrials.gov/ct2/show/NCT03682562). The present study was approved by the research ethics committee of Faculty of Dentistry, Cairo University (approval number 18-9-53) (approval date 26/9/2018) and complies with the declaration of Helsinki. Each patient was informed about the details of the study and a signed consent form was obtained from each patient.

The recruitment period extended from July 2019 to February 2020. The participants were recruited from the diagnostic center at the Faculty of Dentistry and the head and neck outpatient clinic at the National Cancer Institute- Cairo University. A convenience sample of 93 subjects was divided into three groups: 30 patients with oral cancer (oral squamous cell carcinoma; OSCC), 30 patients with OPMDs subdivided into: 21 patients with oral lichen planus and 12 patients with leukoplakia, in addition to 30 healthy volunteers who acted as controls. Eligibility criteria for the control group were: no oral lesions on conventional tactile and visual examination as well as good oral hygiene. All included individuals had no systemic disease and no use of recreational drugs (11).

All the study participants were subjected to conventional examination according to the national institute of dental and craniofacial research (12), while the oral cancer group and those with premalignant lesions were subjected to tissue biopsy to confirm diagnosis. The salivary sample was obtained before tissue biopsy on the same day to avoid disease progression bias. The salivary samples were analyzed without knowledge of their assigned group, and histopathologic assessment was done with no knowledge of the index test results to avoid information bias.

- Sample collection and preparation for qPCR

All the study participants were given a sterile tube containing 5mL of 0.9% normal saline solution which they swished (not gargled) in their mouths for 30 seconds, then spat it back into the tube. The tubes were then properly sealed and labelled with the patient's data. The samples were stored in a holding refrigerator at 4 ºC and DNA was extracted within 24 hours of sample collection.

- Extraction of cfDNA from Saliva

Mouth rinse samples were centrifuged. Only the supernatant was harvested with good care not to touch the pellet to ensure full exclusion of any cellular element.

DNA extraction from saliva was done using QIAGEN (Hilden-Germany) DNA extraction minikit. (Catalog number: 52304), according to the manufacturer’s “Blood and Body Fluid Spin Protocol” (13). The concentration of cf-DNA was determined by measuring the absorbance at 260 nm (A260) using the Thermo Scientific NanoDrop 2000 spectrophotometer. Extracted cfDNA samples were stored at -20°C until time of analysis.

- Quantitative PCR of ALU repeats

For the qPCR of the ALU repeats, we used the primer sets manufactured by CUSBIO (Houston, Texas, USA), Sequences of primers in ALU genes were as follows: ALU115 Forward primer sequence was: 5′ CCTGAGGTCAGGAGTTCGAG-3′. While reverse sequence was: 5′CCCGAGTAGCTGGGATTACA-3′. ALU247 forward primer: 5′GTGGCTCACGCCTGTAATC-3′. While reverse primer sequence used was: 5′ CAGGCTGGAGTGCAGTGG-3′.

The reaction mixture for the qPCR contained 1 μl of DNA template, 0.6 μl of each primer (forward and reverse), 7.8 μl of RNAse free H2O and 10 μl of Thermo Scientific Maxima SYBR Green/ROX qPCR Master Mix (2X) catalogue number (#K0221) (14), resulting in 20 μl of reaction volume. A negative control was included with every plate for quality control.

Real-time PCR amplification was performed using the Applied Biosystem StepOne Real-Time PCR system (Applied Biosystems, Foster City,CA). It started with precycling heat activation of DNA polymerase at 95 °C for 10 min, followed by 40 cycles of denaturation at 95 °C for 15 s, annealing at 60 °C for 30 s, and extension at 72 °C for 30 s. To determine the absolute quantitative amount of DNA in the samples, a standard curve was plotted using serial dilutions of 10 to 0.01 ng/μl of DNA. In this method, concentrations of ALU in DNA samples were quantitated by comparing the CT of the unknown sample against the standard curve.

- Calculation of the DNA integrity index

cfDNA integrity was calculated as the ratio of ALU247 concentration to ALU115 concentration (15). 

- Statistical methods

The sample size was calculated using G*Power version 3.1.9.2 according to the mean Log difference of DNA integrity index (DII) and standard deviation calculated from the confidence interval using the RevMan Calculator (https://training.cochrane.org/resource/revman-calculator) as reported by Jiang *et al*. (16) for both the head and neck squamous cell carcinoma and the normal control group. The sample size was calculated with an effect size of 1.479, level of significance of 0.05 and a power of 80%.

Statistical analysis was performed by SPSS (version 20). Quantitative variables were described as median, 25th and 75th percentiles, and range. Shapiro-Wilk test of normality indicated a non-normal distribution of all the quantitative variables, hence, non parametric tests were used. Kruskal-Wallis Test was used for multiple group comparisons, while Mann-Whitney test was used for comparing two groups. Spearman's rho correlation coefficient (ρ) was used for correlation analysis. For Diagnostic testing, sensitivity, specificity, positive predictive values (PPV), negative predictive values (NPV), were calculated with the 95% confidence limits. ROC curve and the area under the curve (AUC) are reported. Significance level is considered at *P* < 0.05. Two Tailed testing was used throughout the analysis for all statistical tests.

## Results

The clinical characteristics of the study groups are shown in Table 1. The groups were age- and sex- matched. The lesion size varied from 38.6 mm2 to 840 mm2, with a median of 205 mm2 (96-328 mm2). As for the tumor grade, the majority of patients had grade II oral cancer (56.67%), while 33.3% had grade I tumors. Grade III and IV tumors were found in only three patients (10%). Table 2 shows the descriptive values of ALU247, ALU115 and DNA integrity index of the three study groups. Intergroup comparison for each of these indices yielded no statistical significance.

**Table 1:**
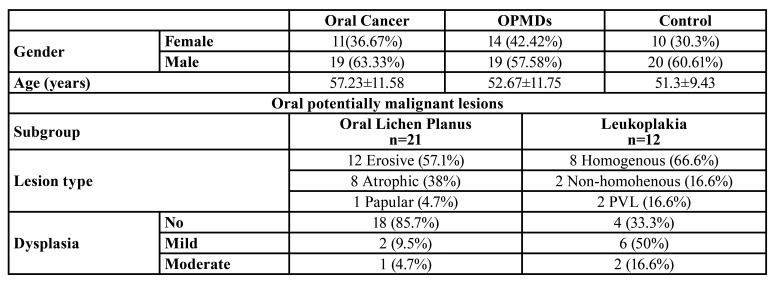
Demographic and clinical characteristics of the study groups.

**Table 2:**
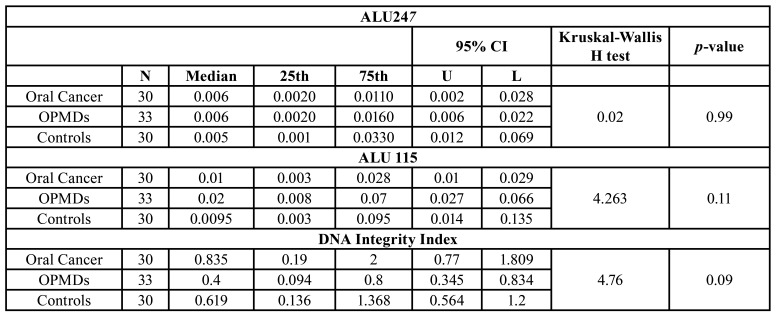
ALU247, ALU115 and DII descriptive values and comparison within the studied groups.

Comparison using the Mann-Whitney test of the subgroups of the OPMD group versus the oral cancer group, revealed a statistically significant higher DII value in oral cancer group as compared to OLP (Table 3), while there was no statistically significant difference between leukoplakia and the oral cancer group. Additionally, leukoplakia had a significantly higher DII level than OLP (Table 3).

Comparison between dysplastic OPMDs and oral cancer revealed higher values in the cancer group but with no statistically significant difference (*p-value*=0.528), nor was there a significant difference between non-dysplastic OPMDs and oral cancer (*p-value*=0.18). Similarly, no statistically significant difference could be found between dysplastic and non dysplastic OPMDs, although the formers showed higher values (*p-value*=0.114)

Additionally, no significant difference was found in-between tumor grades in oral cancer patients with regards to DII (*p-value* = 0.779). There was a weak negative correlation (ρ= -0.18) between lesion size of OLP and leukoplakia and DII, again with no statistical significance (*p-value* =0.3).

**Figure 1:**
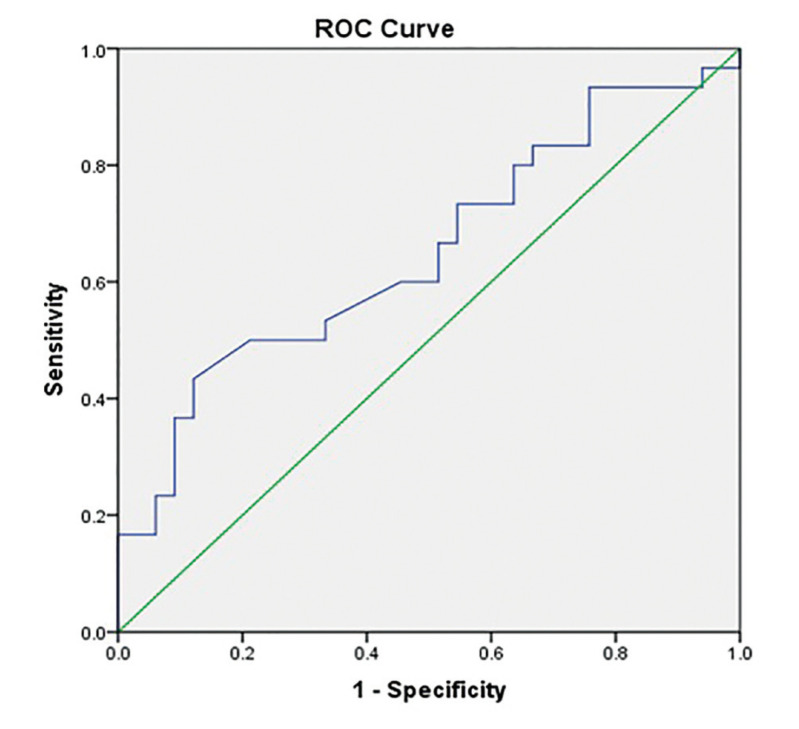
ROC curve plotted between group I and group II.

As the aim of this study was to test DII's ability to differentiate between malignant and potentially malignant lesions, the ROC curve was plotted between oral cancer and OPMD groups (Fig. 1). Youden's J was at a DII value of 1.22 with 40% and 88% sensitivity and specificity respectively. However as DII is intended for screening purposes, an arbitrary cut-off point at 0.236 was used that yielded the most accepTable sensitivity. The AUC was 0.652 (CI: 0.51- 0.78). The diagnostic parameters were 73%, 45%, 55%, 65% sensitivity, specificity, PPV and NPV respectively.

**Table 3:**
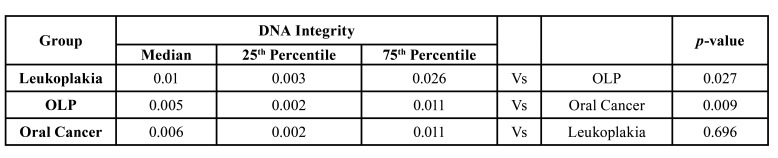
Comparison of DII in leukoplakia, OLP and oral cancer groups.

## Discussion

To the best of our knowledge, the present study is the first to investigate salivary DNA integrity. This phase two diagnostic test accuracy study aimed to assess whether DNA integrity index values can distinguish between a person with and a person without oral cancer among high risk patients with OPMDs.

We found that the DII median value of the oral cancer group was higher than that of the premalignant group. When considered alone, OLP cases had significantly lower DII than both the oral cancer and leukoplakia groups. Oral lichen planus is -by definition- a chronic inflammatory disease (17). Consequently, it is expected to have a high value of DII as reported in other chronic inflammatory diseases such as systemic lupus erythematosus and rheumatoid arthritis (18). However, the results of the present study indicate otherwise. This controversial finding can be justified through basal keratinocyte apoptosis which is a hallmark of the pathogenesis of OLP (17). This increase in apoptosis will lead to an increase in the amount of short DNA fragments which, in turn, increases the value of the denominator in the DII ratio, leading to a smaller DII value.

Conversely, leukoplakia DII did not differ significantly from that of the oral cancer group. This lack of statistical significance could be explained by the histopathology results, where 8 out of 12 patients had dysplasia in the leukoplakia group, while only 3 OLP patients had dysplastic lesions, which may have added to the lower DII values seen in OLP and those higher values found in leukoplakia, approaching the cancer results. Thus, it can be said that DII does not paint all OPMDs with the same brush. In fact, it has the ability to discriminate between OLP and leukoplakia. This positive attribute, is however overshadowed by DII's inability to tell leukoplakia apart from cancer and its failure to differentiate between oral cancer and healthy controls. These factors belie its usefulness as a screening tool for oral cancer.

The present results were in accordance with neither Desai *et al*. nor Jiang *et al*. who investigated DII in oral cancer. Desai *et al*. included a premalignant lesions group and found its DII to differ significantly than the oral cancer group. However, the authors did not specify which premalignant lesions they included in the study nor did they report indices of DII diagnostic accuracy such as sensitivity and specificity (19). Jiang *et al*. found that plasma DII values varied significantly between head and neck cancer patients and healthy controls (16).

 Although no other studies evaluated DNA integrity in oral potentially malignant lesions, there were studies that used an intermediary group diagnosed with a non- cancerous pathosis, while others evaluated DII in different body fluids other than blood. For example, Sriram *et al*. measured pleural fluid DII in malignant and benign effusion and found a statistically significant difference between their values (20)

Stötzer *et al*. evaluated DNA integrity in plasma of patients with metastatic breast tumors, localized malignant and benign tumors, and healthy controls. The benign tumors group scored the lowest values, even lower than the control group. The authors concluded that due to the elevated values of DII in some of the healthy controls, it is unsuiTable for use on an individual case basis (10). Partially similar results are found in the current study where DII in patients with premalignant lesions was lower than in the control group. However the difference was of no statistical significance. Although the present study excluded controls with factors that may affect DNA integrity such as systemic diseases, bad oral hygiene and recreational drug use, there were still many factors that could affect DNA integrity such as physical activity, menopause and obesity (21).

Saliva as a source of genomic DNA has been reported to be comparable to blood in many aspects. Although salivary cfDNA yield and quality have been applauded in the literature (22,23), they have seldom been assessed, and studies using salivary cfDNA did not quantitate it (22,24), which makes comparison with blood derived cfDNA and evaluation of overall performance difficult. cfDNA in general can be challenging to work with as it has a high fragmentation tendency, which may cause the loss of DNA primer binding sites and hinder amplification in PCR assays (25). Pre-analytical errors may also be the cause for cfDNA result inconsistency. Up till now, there are insufficient data on how to handle biologic samples such as saliva, and how to optimize isolation and characterization of cfDNA in blood (21), l*et al*one other bodily fluids.

We found DII to have poor diagnostic accuracy with an AUC of 0.65, 73% sensitivity and 45% specificity. Although Sriram *et al*. and (20). Eltorgoman *et al*. used the same study design and groups, they reported conflicting DII accuracy values where Sriram *et al* reported an AUC of 0.766, 57% sensitivity and 90% specificity, while Eltorgoman *et al*. reported an AUC of 0.97, 92% sensitivity, and 92.6% (26). A systematic review on peripheral blood cfDNA integrity reported a pooled sensitivity and specificity of 74% and 84% respectively. It concluded that DII is a promising non-invasive marker, however, there were clashing results as some studies observed an increase in DII in cancer patients while other studies reported the opposite (27).

The findings in this study indicate that although salivary DII can discriminate between OLP and leukoplakia and does not treat OPMDs as a homogenous group, it does however have low discriminatory ability when it comes to OPMDs and cancer and has an overall poor diagnostic performance. Salivary DII is -therefore- not practical for screening for malignant transformation. Whether DNA integrity is a useful tool in other body fluids in other settings remains unclear. The clinical utility of DNA integrity index will remain vague until a clear understanding of the nature of cfDNA is reached and a full grasp on the intricacies of DNA integrity is achieved.
